# Mechanisms of Immunosuppression in Colorectal Cancer

**DOI:** 10.3390/cancers12123850

**Published:** 2020-12-20

**Authors:** Yang Zhang, Ashwani Rajput, Ning Jin, Jing Wang

**Affiliations:** 1 Department of Cancer Biology and Abramson Family Cancer Research Institute, Perelman School of Medicine, University of Pennsylvania, Philadelphia, PA 19104, USA; zhang10@pennmedicine.upenn.edu; 2 Johns Hopkins Sidney Kimmel Cancer Center, National Capital Region, Sibley Memorial Hospital, 5255 Loughboro Road NW, Washington, DC 20016, USA; arajput2@jhmi.edu; 3 Division of Medical Oncology, Department of Internal Medicine, James Comprehensive Cancer Center, The Ohio State University Wexner Medical Center, Columbus, OH 43210, USA; 4 Department of Cancer Biology and Genetics, James Comprehensive Cancer Center, The Ohio State University Wexner Medical Center, Columbus, OH 43210, USA

**Keywords:** colorectal cancer, immunosuppression, immunotherapy, microsatellite stable, cell surface protein, cytokine, transcriptional factor, metabolic alteration, phosphatase

## Abstract

**Simple Summary:**

More emerging studies are exploring immunotherapy for solid cancers, including colorectal cancer. Besides, checkpoint blockade immunotherapy and chimeric antigen receptor (CAR) -based immune cell therapy have being examined in clinical trials for colorectal cancer patients. However, immunosuppression that leads to the blockage of normal immunosurveillance often leads to cancer development and relapse. In this study, we systematically reviewed the mechanism of immunosuppression, specifically in colorectal cancer, from different perspectives, including the natural or induced immunosuppressive cells, cell surface protein, cytokines/chemokines, transcriptional factors, metabolic alteration, phosphatase, and tissue hypoxia in the tumor microenvironment. We also discussed the progress of immunotherapies in clinical trials/studies for colorectal cancer and highlighted how different strategies for cancer therapy targeted the immunosuppression reviewed above. Our review provides some timely implications for restoring immunosurveillance to improve treatment efficacy in colorectal cancer (CRC).

**Abstract:**

CRC is the third most diagnosed cancer in the US with the second-highest mortality rate. A multi-modality approach with surgery/chemotherapy is used in patients with early stages of colon cancer. Radiation therapy is added to the armamentarium in patients with locally advanced rectal cancer. While some patients with metastatic CRC are cured, the majority remain incurable and receive palliative chemotherapy as the standard of care. Recently, immune checkpoint blockade has emerged as a promising treatment for many solid tumors, including CRC with microsatellite instability. However, it has not been effective for microsatellite stable CRC. Here, main mechanisms of immunosuppression in CRC will be discussed, aiming to provide some insights for restoring immunosurveillance to improve treatment efficacy in CRC.

## 1. Introduction

Colorectal cancer (CRC) is the second leading cause of cancer death worldwide, accounting for approximately 880,000 deaths in 2018 [1]. Approximately 70–80% of patients with stage I–III CRC can be treated with curative surgical resection [2], and the 5-year survival rate for early-stage CRC is approximately 90%. However, the survival rate drops to only 10–15% for metastatic (stage IV) CRC [3]. Patients with stage IV CRC are mainly treated with fluorouracil-based chemotherapies, in combination with epidermal growth factor receptor (EGFR) or vascular endothelial growth factor (VEGF) inhibitors, and the median life expectancy is in the range of 21.3 to 30.1 months [4,5,6,7].

CRC is the most common gastrointestinal (GI) cancer. Compared with other tissues, the gastrointestinal tract is home to an enormous number of commensal bacteria and potential pathogens [8]. Therefore, the immune system in the colon and rectum has to fine-tune the balance of the maintenance of tolerance to self-tissue and immunity against potential pathogens and tumorigenic cells. Disruption of the GI tract’s normal immune homeostasis may cause autoimmunity diseases, including inflammatory bowel disease [9]. In contrast, normal immunosurveillance is critical to eliminate potential pathogens and eradicate sporadic tumorigenic cells to prevent GI diseases, including CRC.

It has been well recognized that the immune system plays a key role in colorectal cancer development and progression [10]. The presence of CD8^+^ T cells in the tumor bed and invasive margin is strongly associated with disease-free survival and overall survival in CRC and the “immune score”, quantifying the density and location of immune cells within the tumor, has provided prognostic value that may be superior to the American Joint Committee on Cancer/Union for International Cancer Control (AJCC/UICC) tumor (T), nodes (N), and metastases (M) (TNM) classification in CRC [11,12]. This indicates that adaptive immune response may play a role in CRC progression [13] and immunotherapy may be promising in CRC treatment. However, the efficacy of immune checkpoint inhibitor monotherapy targeting programmed cell death-1 (PD-1) is only seen in a small subset of patients with microsatellite instability high (MSI-H) status. In contrast, no objective response was observed in microsatellite stable (MSS) CRCs, which account for the majority of the cases [14]. The mechanisms for resistance to anti-PD-1 immune checkpoint blockade in MSS CRC include lack of tumor neoantigens, immune exclusion, and immunosuppression. Higher tumor mutation burden (10- to 50-fold) and more neoantigens in MSI-H tumors, together with higher levels of multiple checkpoints, including PD-1, cytotoxic T lymphocyte-associated antigen 4 (CTLA-4), and lymphocyte activation gene 3 (LAG3) in MSI-H tumors, may explain in part why anti-PD-1 treatment works more efficiently in MSI-H CRCs than in MSS CRCs [15,16]. With the recent advancement in combination strategies and immunomodulatory monoclonal antibodies (mAbs) targeting other immune checkpoints, there is great enthusiasm in developing novel immunotherapies in CRC.

In this review, we will focus on the systemic review of the mechanisms of immunosuppression and the clinical approaches to circumvent immunosuppression in CRC.

## 2. Mechanisms That Mediate Immunosuppression in CRC

### 2.1. Immune Cell-Mediated Immunosuppression

**Figure 1:**
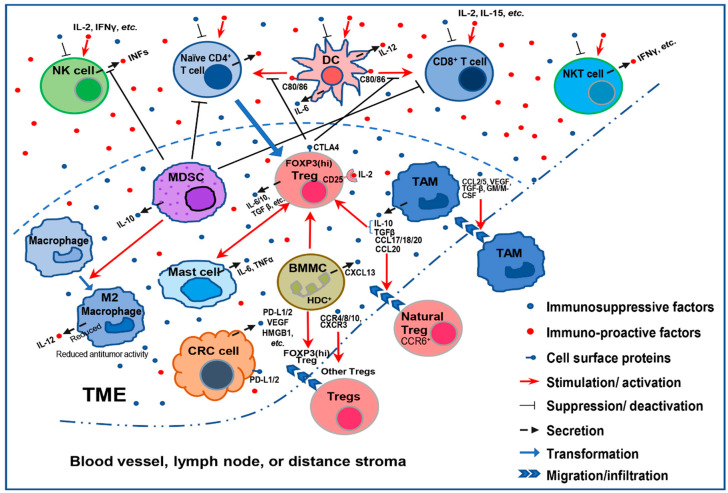
A simplified schematic showing how immunosuppressive cells in the TME assist tumor cells escape from immunosurveillance. In the TME, the immunostimulatory cytokines (IL-2, IFN-γ, IL-15, etc.) may enhance or activate immunity against tumor cells. Nevertheless, immunosuppressive cytokines (PD-L1, TGFβ, etc.) secreted by immunosuppressive cells or cancer cells may inhibit proliferation or activation of naïve CD4^+^ T cells and tumor-killing immune cells, including natural killer (NK) cells, CD8^+^ T cells, and NKT cells. Meanwhile these immunosuppressive cytokines (CCR4, CCL20, TGFβ, etc.) secreted by tumor-associated macrophages (TAMs), mast cells, and bone marrow myeloid cells (BMMCs) may assist tumor infiltration of immunosuppressive cells, such natural T regulatory cells (Tregs) and TAMs, and support proliferation or activation of immunosuppressive cells, such as Tregs. Immunosuppressive cells, including myeloid-derived suppressor cells (MDSCs) and Tregs, may also inhibit the function or activation of NK cells, T cells, and NKT cells independent of cytokines.

In a normal microenvironment, different immune cells are kept in check and balanced to maintain homeostasis. Under the invasion of pathogens or tumor cells, the immune system is reprogrammed to eliminate these invasions, representing an immune-activated status (inflammation). In the tumor microenvironment (TME), a normal immune-activated status is detrimental to the tumor cells, which leads to the eradication of the tumor cells. On the other hand, tumor cells may escape the immune surveillance by changing the cytokines or reprogramming the immune cells to support an immunosuppressive TME and facilitate the initiation and progression of CRC. These immune cells include T regulatory cells (Tregs), tumor-associated macrophages (TAMs), myeloid-derived suppressor cells (MDSCs), cancer-associated fibroblasts (CAFs), mast cells (MCs), and a group of newly identified bone marrow myeloid cells (BMMCs) (Figure 1).

Tregs are essential for maintaining peripheral tolerance, blocking autoimmunity, and limiting chronic inflammatory diseases. Tregs play important roles in tumor initiation and progression by inhibiting antitumor immunity. There are five major Treg-mediated immune suppressive mechanisms: suppression of costimulatory signals by CD80 and CD86 expressed by dendritic cells through CTLA-4, competing with effector T cells for extracellular interleukin (IL)-2 by high-affinity IL-2 receptor—CD25 (IL-2 receptor α-chain); secretion of inhibitory cytokines; metabolic modulation of tryptophan and adenosine; and induction of apoptosis of effector T cells. Tregs are generally attracted to and activated in the TME by chemokine gradients such as CCR4-CCL17/22, CCR8-CCL1, CCR10-CCL28, and CXCR3-CCL9/10/11. A high infiltration by Tregs was considered to be associated with poor survival in many cancers [17]. However, CRCs, which are commonly infiltrated by suppressive forkhead box P3 (FOXP3) Tregs, can be further classified into two types by either a low or high expression level of FOXP3. FOXP3(lo) non-suppressive T cells, characterized by instability of FOXP3, do not express the naive T cell marker CD45RA and secrete inflammatory cytokines, differing from competent FOXP3(+) Tregs. CRCs with an abundant infiltration of FOXP3(hi) Tregs showed significantly worse prognosis than those with predominantly FOXP3(lo) Treg infiltration [18]. Taken together, Tregs, especially FOXP3(hi) Tregs, promote comprehensive immunosuppression in the TME to assist tumor initiation and progression of CRC.

TAMs may mediate systemic immunosuppression in the TME of CRC. TAMs derive from monocytic precursors circulating in blood and are recruited to the tumor site by different molecules, such as the chemokines CCL2 and CCL5, VEGF, TGF-β, and colony-stimulating factors (GM-CSF and M-CSF) [19]. TAMs consist of two major cell subtype populations classified as either M1 or M2 macrophages. M1 macrophage activation is essential in the host defense response to tumor cells, while M2 macrophage activation mediates parasite defense and is associated with wound healing [20]. Generally, TAMs more closely resemble M2 macrophages with Th2 immune responses, which secrete an array of immunosuppressive chemokines, cytokines, and enzymes to exert their functions and downregulate the activation of multiple immune cells. These immunosuppressive factors secreted by TAMs can also directly inhibit the function of CD8^+^ and CD4^+^ T cells, induce the production of Tregs, and recruit natural Tregs into the TME. As a result, a positive feedback loop is formed to further induce immunosuppression in the TME, in which Tregs in return further inhibit effector T cells or secrete more immunosuppressive factors [21,22]. In CRC, by secreting CCL17, CCL18, and CCL22, TAMs recruit naïve and Th2 lymphocytes and induce ineffective antitumor responses in the TME [19]. Additionally, CCL20 secreted by TAMs recruit CCR6^+^ natural Tregs to stimulate immunosuppression in CRC. Conversely, conditional macrophage knockout reduced CCL20 levels, blocked CCR6^+^ natural Treg recruitment, and suppressed tumor growth in a subcutaneous CRC cells (CMT93)/CD11b^−^ diphtheria toxin receptor (DTR) model [23]. Furthermore, immunosuppressive cytokines IL-10 and TGF-β produced by TAMs induce the production of Tregs by upregulating the pivotal regulatory transcription factor FOXP3 in CD4^+^ T cells [24].

The role of MDSCs as critical inhibitory regulatory cells that attenuate antitumor immunity has been well studied. MDSCs reduce antitumor immunity by perturbing innate immunity through their interactions with macrophages, natural killer (NK) cells, and NKT cells. In the presence of MDSCs, macrophages are transformed to an M2 or alternatively activated phenotype with reduced antitumor activity. This re-polarization is due to crosstalk between MDSCs and macrophages, which increases MDSC production of immunosuppressive cytokine IL-10 and decreases macrophage production of immune-provocative cytokine IL-12 [25]. In addition, some subpopulations of MDSCs can suppress NK cell cytotoxicity by blocking NK cell-mediated production of interferons (IFNs). Furthermore, the accumulation of MDSCs and M2 macrophages in the TME can be driven by type II NKT cell-produced IL-13, thereby facilitating tumor progression [26]. For example, the blockade of IL-13 or the elimination of either type II NKT cells or myeloid cells could interrupt this immunosuppressive circuit and restore immunosurveillance, preventing tumor recurrence in a CT26 CRC lung metastasis model [27]. MDSCs can also potently inhibit T cell (both CD4^+^ and CD8^+^) activation, which requires cell-to-cell contact independent of major histocompatibility complex (MHC). In vivo treatment of tumor-bearing (including CRC tumor) mice with agents reducing the level of MDSCs enhanced T cell activation and delayed tumor progression. In contrast, adoptive transfer of tumor-induced MDSCs into tumor-free mice promotes tumor growth and inhibits T cell activation [26]. Moreover, the presence of increased immunosuppressive circulating and tumor-resident Lin^-/low^HLA-DR2CD11b^+^CD33^+^ MDSCs in patients with CRC is positively associated with advanced cancer stage and metastasis [28]. Thereby, MDSC-mediated immunosuppression in the TME of CRC is mainly a result of the inhibition of T cell activation by MDSCs.

CAFs are derived from resident fibroblasts, local pericytes, endothelial cells, epithelial cells, and recruited bone marrow-derived mesenchymal stromal cells (BM-MSCs), depending on the specific type of cancer. CAFs in the TME are involved in modulating the extracellular matrix (ECM), inflammation, cancer cell proliferation, metastasis, angiogenesis, and drug resistance [29,30,31,32] by secreting growth factors, cytokines, and chemokines [33,34]. Notably, CAFs express a pro-inflammatory NF-κB-dependent signature and provide various growth factors to stimulate transformed cells in different tumor models [35,36]. In addition, the stromal gene expression pattern shared with the TGFβ signaling is associated with poor prognosis in CRC patients. Therefore, the use of TGFβ inhibitors blocked the crosstalk between cancer cells and the TME, preventing metastasis formation in the patient-derived tumor organoids [37]. Moreover, there is evidence to suggest that CAFs may reduce CD8^+^ T cell viability in an antigen-specific and -dependent manner via PD-L2 and Fas ligand (FasL) [38]. These studies indicate that CAFs can enhance key immune checkpoints to shield tumor cells from immunity.

MCs may also support an immunosuppressive TME in CRC and promote tumor progression. MCs are tumor-resident myeloid cells. The interaction between MCs and Tregs may trigger either immunosuppression or the loss of function of Tregs and autoimmunity [39]. Studies have shown, in both human CRC and murine polyposis, that the outcome of this interaction is the generation of potently immunosuppressive for antitumor activity, but pro-inflammatory Tregs. These Tregs shut down IL-10, gain the potential to express pro-inflammatory cytokine IL-17, and switch from suppressing to promoting MC expansion and degranulation. In CRC, MCs induce Tregs to shift function and escalate inflammation without losing T cell-suppressive properties [40]. Interestingly, both Tregs and MCs are expressed at high levels in CRC tumors. As evidence of the immunosuppressive role of MCs in the TME of CRC, the density of low tumor-infiltrating MCs confers prognostic benefit and reflects immune activation in CRC [41,42].

Recently, a newly identified distinct subset of dynamic BMMCs [43], which expresses histidine decarboxylase (HDC^+^), has been shown to induce immunosuppression in the TME of CRC. These HDC^+^ BMMCs support tumor infiltration of CD4^+^ FOXP3^+^ Tregs and strongly support colonic tumorigenesis of CRC. Additionally, the HDC^+^ myeloid-derived Cxcl13/Cxcr5 axis has been shown to regulate FOXP3 expression and Treg proliferation. The deletion of HDC^+^ myeloid cells or the disruption of the Cxcl13/Cxcr5 axis by gene knockdown impaired the production and recruitment of Tregs. Overall, HDC^+^ granulocytic myeloid cells affect CD8^+^ T cells directly and indirectly through the modulation of Tregs and thus appear to play key roles in suppressing anticancer immunity [44].

In addition to immune cells, there are other elements that contribute to the immunosuppression of CRC, including cell surface proteins (receptors and enzymes), cytokines/chemokines, etc. (Table 1). We will review their function in the immune regulation of CRC below.

### 2.2. Cell Surface Protein-Mediated Immunosuppression

Immune cells express different cell surface receptors, which is critical for normal immune homeostasis, immune defense of pathogens, and antitumor activity. However, the activation of these receptors may also trigger downstream signaling pathways that lead to immunosuppression in the TME of CRC. Besides, a small group of cell surface enzymes on immune cells can also evoke immunosuppression. The receptors/surface enzymes that play such a dominant role in CRC include PD-1, T cell immunoglobulin and immunoreceptor tyrosine-based inhibitory motif domain (TIGIT), TGFβ receptor II, glycoprotein A repetitions predominant (GARP), CD39, CD73, A2A adenosine receptors, and C-C motif chemokine receptor 8 (CCR8).

The immune checkpoint receptor PD-1, or CD279, can enhance immunosuppression in CRC. PD-1 is one of the co-inhibitory receptors that is expressed on the surface of antigen-activated T cells [94]. Though PD-1 interacts with both PD-L1 and PD-L2 expressed by tumor cells and tumor stroma, only PD-L1 is the dominant inhibitory ligand of PD-1 on T cells in human TME [95,96,97]. The binding of PD-L1 to a PD-1 receptor can activate the downstream signaling in T cells, thus inhibiting the proliferation, cytokine generation and release, and cytotoxicity of T cells. While this immunosuppression will prevent autoimmunity and chronic infection, tumor cells take advantage of it to escape from immune attack. PD-L1-regulated tumor immune resistance includes innate resistance caused by endogenously constitutive PD-L1 expression and adaptive resistance caused by exogenous stimuli-induced PD-L1 expression [95,98]. Interestingly, PD-L1 expression is more prevalent in metastatic CRCs than in primary tumors [45]. Many clinical trials aiming to target PD-1/PD-L1 for immunotherapy in CRC, particularly in metastatic CRC, have yielded some promising outcomes [46]. 

Besides, PD-1 is also expressed in NK cells, TAMs, and activated dendritic cells (DCs) and may contribute to immunosuppression in the TME of CRC [47,48,99]. A study has suggested that PD-1 is overexpressed in peripheral and tumor-infiltrating NK cells of digestive cancers, including CRC, and may suppress NK cell-mediated immunity against tumor cells [47]. Besides, the study of a colon cancer MC38 mouse model has demonstrated that a higher fraction of both the PD-1^+^ TAMs and the PD-1^+^ tumor-infiltrating lymphocytes (TILs) derived from circulating leukocytes contributes to immunosuppression in the TME, and increases tumor burden [48]. Furthermore, PD-1 is also highly expressed in tumor-infiltrated DCs [36]. CRC patients with high levels of tumor-infiltrated DCs exhibited shorter disease-free and overall survival than those with low levels [49]. These studies collectively suggest that PD-1 is broadly expressed in immune cells and mediates a systematic immunosuppression in the TME.

TIGIT, expressed on both T cells and NK cells, is another important checkpoint receptor that facilitates immunosurveillance escape of CRC cells in the TME by promoting T cell and NK cell exhaustion. Checkpoint blockade improves the function of effector T cells or NK cells and has yielded long-term regression in a subset of patients of many cancers. Remarkably, blockade of TIGIT can refresh the antitumor immunity of exhausted cytotoxic T lymphocytes (CTLs) and inhibit tumor growth in a preclinical CRC tumor model [50]. In addition, TIGIT, but not other checkpoint molecules, such as CTLA-4 and PD-1, was linked to NK cell exhaustion in tumor-bearing mice and patients with CRC. Consequently, blockade of TIGIT prevented NK cell exhaustion and enhanced NK cell-dependent tumor immunity in several CRC tumor-bearing mouse models [51]. These studies suggest that TIGIT may be a promising checkpoint receptor to inhibit immunosuppression in the TME of CRC.

TGF-β receptor II is well known for its function in promoting the immunosuppression of T cells. TGFβ signaling is highly implicated in the induction, development, and maintenance of Tregs. Mice with a cell-specific deletion of TGFβ receptor II in T cells showed aberrant T cell activation and increased Th1 and Th2 cell populations. Furthermore, the deletion of TGFβ receptor I on CD4^+^ T cells leads to the disappearance of thymic Tregs during postnatal days 3–5 [52]. Mice with global TGFβ deletion showed similar disease characteristics, and developed a severe, generalized autoimmune disorder and died within 3–4 weeks after birth [53]. Additionally, the number of peripheral Tregs are significantly reduced compared with thymic Tregs in 8- to 10-day-old TGFβ-deficient mice [54]. In fact, TGFβ is required to generate peripheral Treg cells [54] by activating the canonical TGFβ/SMADs signaling; mice with Smad2 and Smad3 deletions on CD4^+^ T cells also developed severe autoimmune disease with reduced FOXP3 expression in the peripheral CD4^+^ T cells [55]. These observations suggest that TGFβ signaling plays an important role in immune surveillance and the suppression of effector T cells through regulating the activation and function of Tregs.

In addition, GARP is another surface receptor expressed by activated Tregs that enhances immunosuppression by binding and activating latent TGFβ signaling. Deletion of GARP in Tregs induces spontaneous inflammation with highly activated CD4^+^ and CD8^+^ T cells and the development of enteritis. Tregs lacking GARP often resulted in pathogenic T cell responses in multiple models of inflammation [100]. GARP^-/-^ Tregs were significantly reduced in specific tissue such as the gut and exhibited decreased expression of colon-specific migratory marker CD103. In the colitis-associated CRC model, GARP on Tregs weaken immune surveillance, and mice with GARP^-/-^ Tregs showed improved protective tumor immunity but did not suppress the growth of xenograft tumor [56]. Though the authors claimed that GARP^-/-^ mice developed less tumors than wild type mice, they did not check cytotoxicity of GARP knockout on the mice [56]. Therefore, more studies are needed to define whether GARP empowers the functionality of Tregs and their tissue-specific accumulation to modulate immunosuppression in CRC.

Cell surface enzyme CD39 on Tregs may also support an immunosuppressive TME through inhibiting effector T cell infiltration of tumor tissue. CD39 is an ectoenzyme mediating hydrolysis of ATP to AMP, which is a rate-determining first step in the generation of immunosuppressive adenosine [101]. Studies have shown that exogenous adenosine significantly reduced the migration of conventional T cells from healthy volunteers. Blocking either adenosine receptors or CD39 enzymatic activity during transmigration restored the ability of conventional T cells from cancer patients to migrate. Adenosine did not directly affect T cells or endothelial cells but prevented monocytes from activating the endothelium [57]. Compared with those of healthy controls, CD39 was expressed at a high level in circulating Tregs or tumor-associated Tregs from CRC patients [57]. These findings suggest that Treg-derived adenosine by CD39 acts as an immunosuppressive factor on monocytes and contributes to reduced transendothelial migration of effector T cells in tumors. Thus, CD39 expressed in Tregs executes its immunosuppressive role by generating adenosine in the TME.

Similar to CD39, another cell surface enzyme CD73 can also suppress immune responses by producing extracellular adenosine. CD73 deletion dramatically inhibited the growth of ovalbumin-expressing CRC MC38 cells. In addition, using an adoptive reconstitution of Treg-depleted DEREG (depletion of regulatory T cells) mouse model, the study also demonstrated that part of the pro-tumorigenic effect of Tregs depended on their expression of CD73. Furthermore, in primary CRC tumors, the CD73 deficiency was linked to an increase in the antigen-specific CD8^+^ T cells in peripheral blood and tumors as well as enhanced production of antigen-specific IFN-γ. Studies in bone marrow chimeras revealed that both hematopoietic and nonhematopoietic expression of CD73 assists tumor cells to escape from immune surveillance [58]. These studies indicate that CD73 expressed by both hematopoietic cells and nonhematopoietic cells can boost an immunosuppressive TME of CRC by producing extracellular adenosine.

Activation of A2A adenosine receptors on T cells may also induce immunosuppression in the TME and support tumorigenesis of CRC. Upon elevated extracellular adenosine, the activation of A2A adenosine receptors inhibit T cell–mediated cytotoxicity, cytokine production and T cell proliferation, and increase T cell anergy in TME [102]. The accumulation of extracellular adenosine is often a result of the phosphohydrolysis of extracellular ATP mostly catalyzed by cell surface enzymes CD39 and CD73 under hypoxia or chronic inflammation conditions as discussed [102]. For example, circulating Tregs or tumor-associated Tregs from CRC patients had higher levels of CD39 than those of healthy controls, implying that a CD39/extracellular adenosine/A2A adenosine receptor cascade may mediate immunosuppression in the TME of CRC [57].

CCR8 is a chemokine receptor expressed principally on Tregs and is critical for CCR8^+^ Treg-mediated suppression of anticancer immunity. CCR8 is predominantly expressed on Tregs and a small portion of Th2 cells, but not on Th1 cells. CCR8^+^ Tregs have been shown to be major drivers of immunosuppression and are critical for the function of Tregs in the TME [103]. Recent independent studies have demonstrated that CCR8 was a specific marker selectively upregulated by tumor-infiltrated Tregs in CRC [59]. Furthermore, monoclonal antibody (mAb) therapy against CCR8 significantly reduced de novo induction and suppressive function of Tregs without affecting CD8^+^ T cells, delayed tumor growth, and improved long-term survival in CRC mouse models [60]. These studies suggest that CCR8 expressed in Tregs is a potent immunosuppressor to mediate immunity evasion of CRC cells.

On the other hand, many receptors expressed on immune cells play an important role in activating normal immune responses against pathogens and tumor cells. Disruption of the normal function of these receptors in immunity often leads to immunosurveillance escape of tumor cells in the TME. These receptors include NKG2D receptor, pattern recognition receptors (PRRs), and CD80.

NKG2D receptor may mediate immunosurveillance evasion of CRC cells. NKG2D belongs to the C-type lectin-like superfamily and is expressed on the surface of NK cells as the primary activating receptor. NKG2D ligands (NKG2DL) expressed in tumor cells may bind to NKG2D receptors and activate NK cells, and induce NK cell-mediated cell death of these tumor cells. However, different mechanisms that inhibit the function of NKG2D receptor/NKG2DL lead to the escape of tumor cells from antitumor immunity [104]. Clinical studies have shown that the ratio of NKG2D-positive NK cells of many cancers, including CRC, may be reduced, which acts as an immunosuppressive mechanism to escape surveillance, and the decrease is associated with poor prognosis and metastasis in these malignancies [61]. Another clinical study has demonstrated that the survival advantage of CRC may differ because of the different expression patterns of two groups of NKG2D ligands (MIC and RAET1G) in the tumor cells, suggesting that some expression signature of NKG2D ligands in tumor cells may inhibit the activation of NK cells and induce an immunosuppressive TME in CRC [62].

PRRs, such as toll-like receptors (TLRs), can also mediate immunosuppression in the TME during the initiation or progression of CRC. Upon the recognition of pathogen-associated molecular patterns (PAMPs) that derive from either infectious bacteria or viruses, innate immune cells expressing TLRs trigger immune responses by activating innate and adaptive immune cells [105]. In particular, PRRs respond to endogenous substances released from tumor cells and stress ligands expressed on the tumor cell surface, initiating host immunity as a defensive mechanism against the growing tumor [106]. As a result, certain alterations in the signal pathway may mediate immunosuppression in CRC. For instance, PRR polymorphisms or TLR4 expression by CRC cells may facilitate evasion from immune surveillance, perhaps through iNOS-mediated immune modulation [63].

Dysregulation of CD80 may be immunosuppressive in the TME and taken advantage of by tumor cells of CRC to escape immunity. Interestingly, CD80 can act as either a cell surface protein or a soluble protein. CD80 is a potent costimulatory molecule in immune cells and critical in activating adaptive immune cells for antitumor immunity. As a cell surface protein, it is expressed not only on dendritic cells (DCs), activated B cells, and macrophages, but also on nonprofessional antigen-presenting cells [107]. CD80 may activate T cells by binding to CD28 or being sequestered by a decoy receptor for CD80—CTLA-4 [108]. A recent study has suggested that epithelial CD80 promotes immune surveillance of colonic preneoplastic lesions and its expression is increased by oxidative stress through signal transducer and activator of transcription 3 (STAT3) in CRC cells [64]. However, the cell surface-expressed CD80 may be reduced in CRC cells, thereby leading to immunosurveillance escape of these tumor cells in the TME.

### 2.3. Immunosuppressive Cytokine-Mediated Immunosuppression

In a normal microenvironment, cytokines secreted by immune cells, epithelial cells, fibroblasts, and other stroma cells play essential roles in immune homeostasis, defense of pathogen, and antitumor activity. While many cytokines are immune provocative, others are immunosuppressive. The escalation of immunostimulatory cytokines under different stimulations often facilitates an active status of immunity against pathogens or tumor cells. On the other hand, the balance of cytokines can be reprogrammed by tumor cells, resulting in an immunosuppressive cytokine-dominated TME, which is a common mechanism for immunosurveillance escape of cancer. The major immunosuppressive cytokines in the TME of CRC include TGFβ, VEGF, IL-6, CXCL3, CXCL4, and high mobility group box-1 (HMGB1).

Cytokine TGFβ is a preeminent immunomodulator, as discussed above [65]. VEGF is another potent immunosuppressive modulator in the TME of CRC, in addition to its crucial role in promoting angiogenesis. VEGF mainly mediates immunosuppression through the inhibition of T cell function, the increase in the recruitment of Tregs and MDSCs, and the suppression of differentiation and activation of DCs in the TME [86]. The study of a CRC MCA26-bearing mouse model has shown that a multiple receptor tyrosine kinase-targeting inhibitor—sunitinib—could reduce the expression of IL-10, FOXP3, PD-1, CTLA-4, and BRAF, while increasing Th1 cytokine (IFN-γ), in isolated tumor-infiltrating lymphocytes, likely due to its function of blocking VEGF receptors. An increase in the proportion of CD4^+^ and CD8^+^ T cells was also seen in tumor-infiltrating lymphocytes [66] of these mice. Additionally, studies of bevacizumab, a monoclonal antibody targeting VEGF for patients with metastatic CRC, and inhibitors targeting the VEGF/VEGFR axis in CRC CT26-bearing mice, suggested that VEGF could promote the proliferation of Tregs. Therefore, VEGF/VEGFR antibodies or inhibitors could reduce the number of Tregs in both patients with metastatic CRC and the mouse model [67]. Furthermore, VEGF produced by CRC cells dramatically inhibited the differentiation and functional maturation of DCs from a CD34^+^ precursor. Mechanistically, VEGF represses the activation of NF-κB in hemopoietic progenitor cells, which is the dominant negative effect of IκB, and subsequently prevent DC differentiation [86]. Together, these studies suggest that VEGF is a dominant immunosuppressive factor for tumorigenesis and progression of CRC.

IL6 is a prominent immunosuppressor expressed in DCs, which may support the escape of tumor cells from the immunosurveillance in the TME of CRC. IL-6, a multipotent cytokine, together with IL-6 receptor-alpha (IL6Ra) and gp130, form a complex signal transducer, to recruit Janus kinase, which phosphorylates STAT3. The function of STAT3 signaling involves proliferation, survival, and differentiation of different cell populations, including immune cells and cancer cells. The activation of the IL-6/STAT3 signaling cascade in DCs may mediate immunosuppression by inhibiting antigen presentation ability and suppressing the subsequent antigen-specific helper and cytotoxic T cell responses in vitro and in vivo in an IL-12-dependent manner [87]. Thereby, in IL-6-deficient mice, metastatic colonization of CRC CT26 cells in the liver was reduced, and the antitumor effector function of CD8^+^ T cells, as well as IL-12 production by CD11^+^ DCs, were augmented. These IL-6-deficient mice also exhibited enhanced IFN-AR1-mediated type I IFN signaling, which upregulated PD-L1 and MHC class I expression on CT26 cells [68].

Additionally, studies have also revealed that IL-6 may also support immunosuppression in the TME by repressing the differentiation of IFN-γ-producing helper T cells and promoting subsequent tumor formation [109,110]. For example, a lack of IL-6 in the TME augmented type-1 immunity, including the induction of antitumor cytotoxic T cells, and inhibited tumorigenesis of a CRC CT26 mouse model in vivo [69]. Furthermore, IL-6 is widely produced and STAT3 is activated in the TME of CRC patients. Besides, the CD11b^+^CD11c^+^ population isolated from tumor tissues shows higher IL-6 expression compared with the same phenotypic population isolated from peripheral blood mononuclear cells (PBMCs), suggesting that T cell receptor (TCR)-mediated activation of both CD4^+^ T cells and CD8^+^ T cells was significantly reduced in these tumor tissues compared with PBMCs [68,69]. While repressing the differentiation of naïve T cells to Tregs, IL-6 stimulates Th17 T cell maturation from naïve T cells [111]. Th17 cells are a key player in the pathogenesis of many immune-related diseases, including cancer [112]. Studies further suggested that the percentage of Th17 cells and the serum IL-6 level are dramatically elevated with aging, and the increase in Th17 T cells and IL-6 levels are correlated with CRC progression, suggesting a pro-tumorigenic role of the IL-6/Th17 T cells axis [113], an effort similar to immunosuppression mediated by IL-6.

Cytokines CXCL3 and CXCL4 may also facilitate immunosuppression in the TME of CRC by regulating Tregs and cytotoxic T lymphocytes (CTLs). CXCL3 is an angiogenic chemokine. CXCL4, also known as platelet factor 4, can be released at high concentrations upon platelet activation to modulate blood coagulation or is maintained at much lower levels in plasma by certain somatic cells, such as lymphocytes [114]. In a syngeneic transplantation tumor model of murine CRC CT26 cells, CXCL4 could be induced by fluorouracil (5-FU) treatment in CRC cells, inhibiting the function of CTLs, and promoting tumor growth in mice [70]. Further study suggested that CXCL4 not only decreased CTL proliferation and IFN-γ production but also enhanced CTL apoptosis and PD-1 expression. Meanwhile, CXCL4 also promoted Treg proliferation and TGF-β production and suppressed PD-1 expression in Tregs. Interestingly, all these immunosuppressive activities by CXCL4 were dependent on CXCL3. Consequently, CXCL4 promotes CRC cell MC38 growth in CXCL4^-/-^ mice and wild type mice, but not in CXCL3^-/-^ mice [71].

Furthermore, cytokine HMGB1 may mediate immunosuppression in the TME by inducing apoptosis of macrophage-derived DCs in CRC. Cytokine HMGB1 secreted by cancer cells has multiple functions in the TME, including inducing apoptosis in macrophages, but promotes cell growth, invasion, and angiogenesis in tumors [115]. HMGB1 reduced the number of mouse peritoneal macrophage-derived dendritic cells (PMDDCs) by inducing apoptosis of PMDDCs, which may be linked to the activation of the (c-Jun N-terminal kinase) JNK pathway. Furthermore, intraperitoneal administration of HMGB1 reduced CD205-positive splenic DCs in C57BL mice. More importantly, both primary and metastasis tumors of metastatic CRC had a higher expression of HMGB1 in lymph node tissues and lower CD205-positive nodal DC numbers than these of primary tumors in non-metastatic CRC. These studies suggest that HMGB1 plays an immunosuppressive role through reducing antigen-presenting PMDDCs in the TME and promotes metastasis of CRC [72].

### 2.4. Immunosuppression Mediated by the Disruption of Anticancer Cytokines

In addition to immunosuppressive cytokines, cytokines that provoke immunity against pathogens or tumor cells act as important immunomodulators against diseases, including cancer. However, dysregulation of these cytokines may induce immunosuppression in the TME and promote tumorigenesis and progression of CRC. Cytokines that may be dysregulated in cancer include IL-12, IL-15, IL-24, CXCL9, CXCL10, and CXCL11.

IL-12 expressed by antigen-presenting cells has been shown to enhance the cytotoxic activity of NK cells and activated T cells, induce the production of IFN-γ, and promote the differentiation of uncommitted T lymphocytes to T helper type 1 cells [116]. An intratumoral injection of adenovirus-expressing IL-12 led to cancer regression of CRC by inducing antitumor immunity against and the tumor cells. Thereby, a reduced level of IL-12 in the TME may facilitate immunosurveillance escape of tumor cells. Indirect evidence suggests that the loss of IL-12 may be immunosuppressive as the level of IL-12 was almost undetectable in the serum of patients with CRC [73].

IL-15, an autocrine/paracrine cytokine produced by tumor cells, has been shown to promote the proliferation, motility, and invasiveness of CRC cells as well as increase their tolerance and resistance to apoptosis. Interestingly, IL-15, as an immunomodulator, is constitutively expressed by DCs, macrophages, fibroblasts, and epithelial cells [117]. Contradictory to its role in promoting tumor progression of CRC, multiple studies suggest that IL-15 could execute its antitumor function through enhancing NK and CD8^+^ T cell cytotoxicity [118]. In a colitis-associated colon carcinogenesis (CAC) model, IL-15 deficiency suppressed NK and CD8^+^ T cell immunity, provided a tumor-supporting inflammatory milieu, and increased tumor burden [74]. Thus IL-15 deficiency can be immunosuppressive in the TME of CRC and assist tumor progression.

IL-24 can act as an immunological regulatory cytokine to mediate the induction of Th1 cytokines. Recent studies have suggested that adenovirus-mediated IL-24 may also potentially enhance immunosurveillance against tumor cells by increasing the levels of CD8^+^ T cells in mammary tumors and fibrosarcoma, thereby contributing to tumor suppression in mice [75]. In a murine model of CRC, IL-24 caused CD4^+^ T cells and CD8^+^ T cells to secrete IFN-γ and enhanced the cytotoxicity of CD8^+^ T cells in vivo. More importantly, IL-24 transformed the TME and enhanced anti-tumor effects in favor of tumor elimination [76]. Additionally, IL-24 expression correlated inversely with the clinical stage of human CRC, further suggesting an immune activator role of IL-24 [76]. Consequently, an immunosuppressive TME could be a result of low levels of IL-24. For example, CRC tissues displayed significantly lower IL-24 levels compared with the adjacent mucosa [77].

The IFN-regulated CXC chemokines, CXCL9, CXCL10, and CXCL11, serve as T cell attractants and can be produced in response to cytokine stimulation. Evidence from the engineered expression of CXCL10 in isogenic rectal CT26 cancer cells suggests that they provide a protective and anti-metastatic role, mediated mainly by adaptive immunity of tumor-infiltrating T cells [78]. In addition, the expression of CXCL9, CXCL10, and CXCL11 was significantly correlated with the presence of tumor-infiltrating cytotoxic CD8^+^ T cells and CD4^+^ TH1 effector cells, which represent an independent positive predictor of prolonged disease-free survival of CRC patients [78,79]. Reduced production of CXCL10 may give rise to an immunosuppressive TME. A study from the CRC APC^min/+^ mouse model suggested that Tregs can inhibit endothelial CXCL10 production, inhibit T cell migration in tumors, and that CXCR3 (receptor of CXCL9, CXCL10, and CXCL11)-mediated signaling is crucial for lymphocyte accumulation in intestinal tumors [80]. Collectively, even though these IFN-regulated CXC chemokines can evoke adaptive immunity against tumor cells, the expression reduction of these chemokines by different mechanisms (e.g., Treg-mediated repression of CXC chemokines in endothelial cells) may support an immunosuppressive microenvironment in CRC by inhibiting T cell infiltration of tumor tissues.

### 2.5. Phosphatase-Mediated Immunosuppression

SHP2 may act as an immunosuppressive factor in CRC by suppressing T cell activation. SHP2 is a non-receptor ubiquitous protein tyrosine phosphatase, which can be activated by PD-1 [81]. In CD4^+^ T cells, dephosphorylation of STAT1 and STAT3 by SHP2 results in their inactivation and inhibition of their downstream signal transduction for the secretion of IFN-γ (by STAT1) and production of IL-17A (by STAT3), both of which are critical for T cell activation [82]. Consequently, in CD4^+^ T cells with SHP2 knockout, STAT1 is hyper-activated, triggering increased Th1 differentiation and IFN-γ production, which further enhances the activity of CD8^+^ CTLs for anti-tumor immunity [83]. Thereby, SHP2 deficiency in T cells inhibits the development of colitis-associated colorectal cancer and the growth of xenograft tumors of CRC [81,83]. Interestingly, combination treatment with an inhibitor of SHP2 and an anti-PD-1 antibody generates a synergistic inhibitory effect on the growth of CT-26 cells and MC-38 cell xenograft tumors [81]. Taken together, these studies suggest that SHP2 could be a target to reduce immunosuppression in the TME of CRC.

### 2.6. Transcriptional Factor-Mediated Immunosuppression

Transcriptional factors, including STAT1, STAT3, and NF-κB, may act as downstream targets of immunosuppressive modulators such as IL-6, SHP2, and VEGF to support immunosuppression, as discussed [83,86,87]. Interestingly, STAT3 in CRC cells may also suppress immunosuppression by increasing epithelial CD80 upon oxidative stresses [64]. Therefore, the disruption of STAT3 in CRC cells may result in immunosuppression.

Other transcription factors have also been shown to be involved in immunosuppressive functions due to their essential roles in regulating the expression of important immune regulators. Polycomb repressive complex 2 (PRC2), which mediates epigenetic repressive machinery [119], may suppress anticancer immunity through inhibiting the trafficking of immune cells, including Th1 and CD8+ T cells, to the TME. PRC2 is a chromatin-associated methyltransferase catalyzing the mono-, di-, and trimethylation of lysine 27 on histone H3 (H3K27) [119]. Studies have demonstrated that PRC2 components and demethylase Jumonji domain-containing protein D3 (JMJD3)-mediated histone H3 lysine 27 trimethylation (H3K27me3) suppressed the expression and subsequent production of Th1-type chemokines CXCL9 and CXCL10, mediators of effector T cell trafficking. Moreover, the expression levels of PRC2 components, including the enhancer of zeste homologue 2 (EZH2), polycomb repressive complex 2 subunit (SUZ12), and polycomb protein EED, were inversely correlated with those of CD4, CD8, and Th1-type chemokines in human CRC tissues. This inverse correlated expression pattern was significantly associated with CRC patient survival [84]. These findings suggest that PRC2-mediated inhibition of effector T cell trafficking to the TME is one of immunosuppression mechanisms in CRC.

Forkhead box transcription factor M1 (FOXM1) may negatively regulate immunity against CRC cells by repressing maturation of antigen-presenting bone marrow-derived dendritic cells (BMDCs). FOXM1 plays important roles in tumor initiation and progression, angiogenesis, drug resistance, and metastasis of many cancers, including CRC [120]. In a xenograft model of CRC CT-26 cells, upregulation of FOXM1 by H3K79me2 hindered maturation phenotypes of BMDCs and suppressed BMDC function in a Wnt5a signaling-dependent manner. Furthermore, the upregulation of FOXM1 also decreased T cell proliferation and IL-12 p70 levels. As a result, the repression of FOXM1 by inhibitors of H3K79me2 reduced immunosuppression in the TME and caused xenograft CT-26 tumor regression [85]. Collectively, these studies suggest that FOXM1 induces an immunosuppressive TME by inhibiting antigen-presenting BMDCs in CRC.

Shelterin protein telomere repeats binding factor 2 (TRF2) may enhance immunosuppression by inhibiting NK cell-mediated immunity and support tumorigenesis of CRC. Besides its function as a key factor that maintains telomere integrity in mammals [121], TRF2 in tumor cells impairs their ability to recruit and activate NK cells. Specifically, TRF2 enhances the expression of the HS3ST4 gene, a key modulator inhibiting a cell-extrinsic program that leads to NK cell activation. Consistently, a progressive upregulation of TRF2 is correlated with decreased NK cell density during the early development of human CRC. These studies raise an intriguing possibility that, by assisting tumor cells to dodge innate immune surveillance, overexpression of TRF2 promotes human tumorigenesis [88].

### 2.7. Metabolic Alteration-Mediated Immunosuppression

Metabolic enzyme ornithine decarboxylase (ODC) may provoke immunosuppression by inhibiting M1 macrophage activation and support colitis-associated colon carcinogenesis (CAC). ODC, which is the rate-limiting enzyme for polyamine biosynthesis, controls M1 macrophage activation for the host defense response against tumor cells in gastrointestinal (GI) infections [20,122]. As an example, colitis tissues of mice with myeloid-specific deletion of ODC had increased levels of multiple pro-inflammatory cytokines/chemokines and expression of M1 but not M2 markers. Furthermore, in the azoxymethane (AOM)-dextran sulfate sodium (DSS) model of CAC, myeloid-specific deletion of ODC in mice reduced tumor number, burden, and high-grade dysplasia. These tumors also had expanded M1, but not M2 macrophages [89]. Thereby, ODC may act as an immunosuppressive factor by modulating M1 macrophage activation in the TME of CRC.

Another metabolic enzyme, indoleamine-pyrrole 2,3-dioxygenase 1 (IDO1), may mediate systemic immunosuppression in the TME of CRC. IDO1, an inducible enzyme that catalyzes the rate-limiting step in tryptophan metabolism to produce kynurenine, is generally produced in response to the pivotal immune regulatory Th1 cytokine IFN-γ in various myeloid lineage-derived cells, including DCs and macrophages, as well as endothelial cells, mesenchymal stromal cells, and fibroblasts. TAMs may cause metabolic starvation of T cells, thereby repressing T cell function through the IDO1/2 pathway-generated immunosuppressive metabolites [123]. Both intracellular and extracellular IDO1 in the microenvironment can suppress the function of T cells, and IDO1 is also critical for the production and function of Tregs. Interestingly, stress response kinase GCN2 is required for both the anergizing effect of IDO1-mediated tryptophan depletion on T cells and IDO1-induced activation of Tregs. Comparably, studies have shown that tryptophan catabolites blocked T cell activation and triggered T cell apoptosis, while promoting IDO1-induced differentiation of CD4^+^ cells into Tregs (through a TGF-β-dependent mechanism), with apparent synergistic effects of this effector arm with tryptophan deprivation. Indeed, IDO1 is overexpressed in CRC and correlates with a poor prognosis in patients [90]. These findings suggest that IDO1-mediated metabolic programs assist in shaping an immunosuppressive TME of CRC.

IDO1 is also critical for suppressing the function of NK cells and the production and function of MDSCs. In antigen-presenting DCs, IDO1 is upregulated by IFNs, TLR ligands, and other important immune signals. Notably, IDO1 expression in a small minority population of DCs enables them to dominantly suppress effector T cell responses [124]. Additionally, a recent study further showed that knockdown of IDO1 by shRNAs mainly increased tumor infiltration of neutrophils and delayed tumor progression in a xenograft model of CT26 and MC38 murine CRC cells [125]. Furthermore, IDO is over-expressed in many malignancies, including CRC, and increased expression of IDO correlates with poor prognosis in patients [90,91]. Collectively, these studies indicate that IDO1 may act as a systemic immunosuppressor supporting tumorigenesis and the progression of CRC.

### 2.8. Tissue Hypoxia-Mediated Immunosuppression

Many other factors in the TME also profoundly impact the trafficking or function of immune cells, cytokine level, or characteristics of tumor cells, thereby leading to immunosuppression, among which adenosine and hypoxia are well studied.

The extracellular adenosine level produced by cell surface enzymes, such as CD39/CD73, is an immunosuppressive factor leading to a systematic immune reprogramming for tumor cell evasion of antitumor immunity in the TME, as discussed above [57,58,126].

Hypoxia profoundly reprograms immunity and supports immunosuppression in the TME of CRC. Hypoxia impacts the antitumor immune response by promoting local immune suppression and inhibiting cytotoxicity of immune cells against tumor cells. Hypoxic zones in solid tumors, including CRC, often recruit many immunosuppressive cells, such as Tregs, MDSCs, and TAMs. Hypoxia also potentiates the immunosuppressive function of Tregs and reduces the maturation and function of DCs in the TME [92]. Under hypoxic stress and in the presence of TGF-β, CD4^+^ T cells upregulate FOXP3 through direct binding of hypoxia-inducible factor (HIF)-1 to the FOXP3 promoter region, inducing Treg formation. In a colitis-associated CRC mouse model, severe hypoxia in the colon was accompanied by a reduced differentiation of CD4^+^ effector T cells and an increase in Tregs under hypoxia [93].

## 3. Current Immunotherapy Trials for MSS CRC

To reverse the immunosuppressive TME and enhance antitumor immunity, several strategies have been attempted (Table 2), including (1) suppression of the cells or signaling pathways that promote immunosuppression in TME; (2) engagement of immunostimulatory cells through external stimulations or treatments; and (3) replacement of the exhausted immune cells with engineered re-activated immune cells. Of course, the combination strategy may achieve the most promising result to deter immunosuppression in the TME.

While anti-PD-1/PD-L1 therapies have demonstrated great efficacy in CRC patients with microsatellite instability high/deficient mismatch repair (MSI-H/dMMR), monotherapy is not effective in patients with advanced MSS CRC [14]. The combination strategy of anti-PD-1 or anti-PD-L1 with anti-CTLA-4 has been investigated to enhance antitumor immune response. Durvalumab (anti-PD-L1) plus tremelimumab (anti-CTLA-4) showed moderate improvement in overall survival (OS) in patients with MSS CRC who were previously treated with chemotherapies without a significant *p* value (hazard ratio = 0.7, *p* = 0.07). There is no difference in progression-free survival (PFS) in the immunotherapy group compared to the best supportive care group [132].

VEGF-targeting therapies, such as bevacizumab, have been shown to attenuate the tumor-induced immunosuppressive microenvironment by decreasing the number of Tregs in both pre-clinical mouse models and patients with CRC [67,86]. However, adding atezolizumab (anti-PD-L1) to fluorouracil and bevacizumab as a first-line maintenance treatment for patients with metastatic MSS CRC did not result in improvement in efficacy in MSS CRC [130].

The alternative strategy is to block immunosuppressive cytokines that mediate the recruitment of Tregs, MDSCs, and TAMs. For example, TGF-β contributes to the immune exclusion in the TME at later stages of tumor development. Although TGF-β blockade as monotherapy is disappointing [133], the combination treatment of a small-molecule inhibitor (galunisertib) against TGF-β with anti-PD-1 or anti-PD-L1 agents has being tested in clinical trials of solid tumors (NCT02423343 and NCT02734160) and will likely yield promising outcomes. In addition, clinical trials with anti-PD1/PD-L1 in combination with anti-CD73, anti-adenosine A2A receptor, or triplet therapy are being examined in MSS CRC (NCT02503774, NCT03207867, and NCT03549000). The efficacy and safety of a STAT3 inhibitor (BBI608) in combination with pembrolizumab are also being assessed in a phase Ib/II study for patients with metastatic MSS CRC (NCT02851004).

Chemotherapy, such as FOLFOX, has been shown to induce immunogenic cell death, increase antigen presentation and activate PD-1^+^ CD8^+^ T cells [134,135]. Therefore, immune checkpoint inhibitors (ICIs) in combination with standard-of-care chemotherapies may potentiate ICI efficacy by promoting a more immunogenic TME. In a phase II study, clinical activity was seen in the combination treatment of embrolizumab (anti-PD-1) with FOLFOX for patients with untreated advanced CRC, including those with proficient MMR (70%), even though the FOLFOX dose was reduced due to increased neutropenia in the initial cohort [136]. Besides, preliminary efficacy data showed a 70% objective response rate (ORR) for the combination treatment of pembrolizumab with FOLFOX in metastatic CRC patients with MMR-proficient disease in the first-line setting of an ongoing clinical trial (keynote-651 cohort B, NCT03374254) [137].

To target the immune-excluded TME in MSS CRC, a new strategy is to recruit T cells into the immunosuppressive TME by using T cell bispecific (TCB) antibodies. Bispecific antibody is designed to simultaneously bind two different epitopes or antigens, physically linking two binding specificities that may be temporally or spatially separate [138]. CEA-TCB (RG7802, RO6958688), a novel bispecific antibody that simultaneously binds to carcinoembryonic antigen (CEA) on tumor cells and CD3 on T cells, selectively engages effector T cells to kill CEA-expressing tumor cells. It was investigated in combination with atezolizumab in a phase 1 trial in patients with MSS CRC. Antitumor activity was observed during dose escalation with CEA-TCB monotherapy, with increased intratumoral CD3 T cell infiltration. Enhanced activity and a manageable safety profile were seen in combination with atezolizumab [131].

Besides the immune checkpoint inhibitors, adoptive cell transfer (ACT) has been exploited to enhance the activity of immune effector cells in the TME. Cytotoxic T cells and NK cells are the major effector cells in the anti-tumor response. Compared to other strategies, ACT has several advantages, including the allowance of ex vivo expansion of tumor-specific lymphocytes and the infusion of cells that can be genetically engineered. In a phase I/II study, sentinel lymph node (SLN)-T lymphocytes were expanded ex vivo and then transfused to CRC patients who underwent radical or palliative surgery. The 24-month survival rate of the SLN-T lymphocyte group was significantly higher than that of the control group (55.6% versus 17.5%, *p* = 0.02). This study showed that SLN-T lymphocyte immunotherapy was feasible and safe and may improve OS in metastatic CRC [127].

Chimeric antigen receptor (CAR)-T cell therapy uses chimeric T cell receptors encompassing an extracellular antigen-binding domain, transmembrane hinge domain, and an intracellular signaling domain with costimulatory molecules (such as CD28) in tandem with a CD3ζ chain. The CAR thereby reactivates the T cell immunity against tumor cells. However, CAR-T cell therapy has limited efficacy for the majority of solid tumors, due to limited trafficking and persistence of T cells into the tumor and an immunosuppressive TME [128]. Recently, a novel NKG2D CAR-T cell therapy, which uses a non-viral third-generation NKG2D CAR, yielded a promising outcome when tested for its antitumor activity against human CRC cells in vitro and in vivo [129]. To test the notion in a clinical trial, a phase I study is evaluating the safety and clinical activity of NKG2D CAR-T cells in combination with neoadjuvant FOLFOX in patients with metastatic CRC whose liver metastases are potentially resectable (NCT03310008). Additionally, NK cell-based cell therapy, which uses chimeric antigen receptor-expressing NK cells from the patient for anticancer therapy, has also made it to clinical trials for solid tumors, including CRC (NCT02839954) [139,140]. Further investigations will provide a more definitive conclusion if CAR-T cell therapies can benefit patients with MSS CRC.

## 4. Conclusion: Limitations and Future Directions 

Due to the intricate nature of the TME and the interplay of immunity and tumor cells, our knowledge on immunosuppression that leads to cancer immune escape remains limited. The mechanisms discussed in this review mainly focus on the regulation of immunosuppression in the TME of CRC, especially highlighting the possible clinical relevance and implication. While some cells, cytokines, receptors, and signaling pathways may provoke an immunosuppressive TME to promote tumor initiation and progression, others may act as immune activators to enhance immunity against tumor cells. Thereby, instead of one or a few immunosuppressive factors in the TME, the balance of immunosuppressive and antitumor immune-provocative factors may ultimately determine the impact of the microenvironment on tumor cell survival and proliferation. Although it may still be challenging even with advanced high-throughput DNA/RNA sequencing, comprehensive metabolic/proteomics analytic platforms, powerful multicolor flow cytometry (cell analyzers), and bioinformatics tools, a systematic analysis of the signatures and profiling of tumor cells and the TME will not only provide useful information to understand the immunosuppression in the TME of CRC but also help determine the most comprehensive and promising treatment plan for patients with CRC.

## Figures and Tables

**Table 1:** Factors that contribute to immunosuppression in the TME of CRC.

Classes	Immunosuppressors	Major Immune Cells Affected	References
**Cell surface protein**	PD-1	T cell, NK cell, TAM, DC	[45,46,47,48,49]
	TIGIT	T cell, NK cell	[50,51]
	TGFβ RII	Treg	[52,53,54,55]
	GARP	CD4^+^ and CD8^+^ T cells	[56]
	CD39	NK cell	[57]
	CD73	Effector T cell	[58]
	A2A adenosine receptors	T cell	[57]
	CCR8	Treg	[59,60]
	**Disrupted Immunosurveillance** **Contributors**	**Major Immune Cells Affected**	**References**
	NKG2D receptor	NK cell	[61,62]
	TLRs	Innate immune cell	[63]
	CD80	T cell	[64]
**Cytokines/Chemokines**	**Immunosuppressors**	**Major Immune Cells Affected**	**References**
	TGFβ	Treg	[65]
	VEGF	T cell, MDSC, DC	[66,67]
	IL-6	CD4^+^ and CD8^+^ T cells	[68,69]
	CXCL3 and CXCL4	Treg, CD8^+^ T cell	[70,71]
	HMGB1	PMDDC	[72]
	**Disrupted Immune Activators**	**Major Immune Cells Affected**	**References**
	IL-12	NK cell, T cell	[73]
	IL-15	NK cell, CD8^+^ T cell	[74]
	IL-24	CD4^+^ and CD8^+^ T cells	[75,76,77]
	CXCL9, CXCL10, and CXCL11	CD4^+^ and CD8^+^ T cells	[78,79,80]
**Phosphatase**	**Immunosuppressor**	**Major Immune Cells Affected**	**References**
	SHP2	CD4^+^ and CD8^+^ T cells	[81,82,83]
**Transcriptional factors**	**Immunosuppressors**	**Major Immune Cells Affected**	**References**
	PRC2	Th1 and CD8^+^ T cells	[84]
	FOXM1	BMDC	[85]
	NF-κB	DC	[86]
	STAT1	CD4^+^ T cell	[83]
	STAT3	CD4^+^ T cell, DC	[82,87]
	TRF2	NK cell	[88]
**Metabolic enzymes**	**Immunosuppressors**	**Major Immune Cells Affected**	**References**
	ODC	M1 macrophage	[20,89]
	IDO1	CD4^+^ and CD8^+^ T cells, NK cell, MDSC	[90,91]
**Other factors**	**Immunosuppressive factors**	**Major Immune Cells Affected**	**References**
	Extracellular adenosine level	T cell	[57,58]
	Hypoxia	Treg, CD4^+^ effector T cell, MDSC, TAM, DC	[92,93]

Abbreviations: dendritic cell, DC; tyrosine-based inhibitory motif domain, TIGIT; high mobility group box-1, HMGB1; peritoneal macrophage-derived dendritic cells, PMDDC; ornithine decarboxylase, ODC.

**Table 2:** Current strategies for immunotherapy for CRC.

Strategies	Drugs/Therapy	Drug Target/Action	Note
**Blocking cells or signaling pathways that mediate immunosuppression**	Pembrolizumab	PD-1	FDA-approved
	Durvalumab	PD-1	FDA-approved
	PDR001	PD-1	Not tested alone in CRC
	Atezolizumab	PD-L1	FDA-approved for other cancers
	Tremelimumab	CTLA-4	Not tested alone in CRC
	Bevacizumab	VEGF	Improve survival of CRC patients
	Galunisertib	TGFβ	Not effective alone
	BBI608	STAT3	Not tested alone in CRC
	Oleclumab	CD73	Not tested alone in CRC
	NZV930	CD73	Not tested alone in CRC
	NIR178	Adenosine A2A receptor	Not tested alone in CRC
**Engagement of immunostimulatory cells through external stimulations or treatments**	FOLFOX	Increase antigen presentation and activate PD-1+ CD8+ T	Standard care for advanced CRC
	CEA-TCB	Recruit T cells to TME/tumor cells	(RG7802, RO6958688)
**Replacement of the exhausted immune cells with engineered re-activated immune cells**	Sentinel lymph node (SLN)-T lymphocytes ACT	Replace exhausted cytotoxic T cells	May improve OS in metastatic CRC [127]
	(CAR-) T cell therapy	Replace exhausted T cells	Limited efficacy in solid cancers [128]
	NKG2D CAR-T cell therapy	Replace exhausted T cells	Promising outcomes in preclinical study [129]
	(CAR)-NK cell therapy	Replace exhausted NK cells	NCT02839954
**Combination strategy**	Durvalumab + tremelimumab	Target PD-1 and CTLA-4	Moderate improvement in OS for MSS CRC patients
	Bevacizumab + fluorouracil	Target VEGF and increase antigen presentation and activate PD-1^+^ CD8^+^ T	First- and second-line treatment of metastatic CRC
	Atezolizumab + fluorouracil + bevacizumab	Target PD-L1 and VEGF, and increase antigen presentation and activate PD-1^+^ CD8^+^ T	No further improvement in efficacy in MSS CRC [130]
	Pembrolizumab + FOLFOX	Target PD-L1 and increase antigen presentation and activate PD-1^+^ CD8^+^ T	NCT03374254
	Atezolizumab + TCB-CEA	Target PD-L1 and recruit T cells to TME/tumor cells	Increased antitumor activity [131]
	Pembrolizumab + BBI608	Target PD-L1 and STAT3	NCT02851004
	Durvalumab + oleclumab	Target PD-1 and CD73	NCT02503774
	PDR001 + NIR178	Target PD-1 and adenosine A2A receptor	NCT03207867
	PDR001+ NZV930 + NIR178	Target PD-1, CD73, and adenosine A2A receptor	NCT03549000
	NKG2D CAR-T cell therapy + FOLFOX	Increase antigen presentation and activate PD-1^+^ CD8^+^ T, and replace exhausted T cells	NCT03310008

Abbreviations: Folinic acid (leucovorin), Fluorouracil (5-FU), and Oxaliplatin (Eloxatin), FOLFOX; Carcinoembryonic Antigen T-Cell Bispecific antibody, CEA-TCB; adoptive cell transfer, ACT; overall survival, OS.
